# The diagnostic accuracy of screening for psychosis spectrum disorders in behavioral health clinics integrated into primary care

**DOI:** 10.1016/j.schres.2024.02.007

**Published:** 2024-02-28

**Authors:** Mark Savill, Rachel L. Loewy, Tara A. Niendam, A. Jonathan Porteus, Adi Rosenthal, Sarah Gobrial, Monet Meyer, Khalima A. Bolden, Tyler A. Lesh, J. Daniel Ragland, Cameron S. Carter

**Affiliations:** a University of California, Davis, United States of America; b University of California, San Francisco, United States of America; c WellSpace Health, United States of America

**Keywords:** Clinical high risk, Integrated behavioral health, Assessment, Prodromal questionnaire, PQ-B

## Abstract

Screening for psychosis spectrum disorders in primary care could improve early identification and reduce the duration of untreated psychosis. However, the accuracy of psychosis screening in this setting is unknown. To address this, we conducted a diagnostic accuracy study of screening for psychosis spectrum disorders in eight behavioral health services integrated into primary care clinics. Patients attending an integrated behavioral health appointment at their primary care clinic completed the Prodromal Questionnaire - Brief (PQ-B) immediately prior to their intake assessment. This was compared to a diagnostic phone interview based on the Structured Interview for Psychosis Risk Syndromes (SIPS). In total, 145 participants completed all study procedures, of which 100 screened positive and 45 negative at a provisional PQ-B threshold of ≥20. The PQ-B was moderately accurate at differentiating psychosis spectrum from no psychosis spectrum disorders; a PQ-B distress score of ≥27 had a sensitivity and specificity of 71.2 % and 57.0 % respectively. In total, 66 individuals (45.5 %) met criteria for a psychosis spectrum disorder and 24 (16.7 %) were diagnosed with full psychosis, indicating a high prevalence of psychosis in the sample. Overall, screening for psychosis spectrum disorders in an IBH primary care setting identified a relatively high number of individuals and may identify people that would otherwise be missed. The PQ-B performed slightly less well than in population-based screening in community mental health settings. However, the findings suggest this may represent an effective way to streamline the pathway between specialty early psychosis programs and primary care clinics for those in need.

## 1. Introduction

The primary care setting is frequently the first healthcare contact on the pathway to appropriate care amongst people with psychosis (Addington et al., 2002; Cole et al., 1995). However, patients in contact with their primary care provider can experience referral delays to early psychosis services up to twice as long as those who are not receiving primary care services (Anderson et al., 2013a, 2013b). This is problematic, given a longer duration of untreated psychosis (DUP) is associated with poorer outcomes (Kane et al., 2016; Perkins et al., 2005).

Effective early identification and direct referral from primary care, as a frequent first-contact service, could reduce the period between initiating help seeking and receiving appropriate care. This is significant, given this period, known as “supply-side DUP” (Srihari et al., 2014), typically represents the greatest contribution to DUP overall (Birchwood et al., 2013). Symptom checklists in primary care have been explored as a method to improve psychosis detection (French et al., 2012; Woodberry et al., 2022). However, the general mental health screening tools used in earlier efforts resulted in low specificity (French et al., 2012). Since then, the Prodromal Questionnaire Brief (PQ-B; Loewy et al., 2011) has been validated in various settings as a screening tool for psychosis spectrum disorders, which include full threshold psychotic disorders and individuals with attenuated positive symptoms indicative of increased risk of developing a psychotic illness. These settings include early psychosis program referrals (Loewy et al., 2011), community mental health clinics (Niendam et al., 2023), prison populations (Jarrett et al., 2012), schools (Howie et al., 2020), and in the general population using the PQ-B hosted online (McDonald et al., 2019). However, the diagnostic accuracy and the appropriate clinical cutoff threshold has not been explored in primary care generally, nor behavioral health departments integrated into the primary care setting. This is significant, since a different case-mix of participants and illness prevalence can lead to spectrum bias (Willis, 2008), impacting diagnostic test performance. Given the anticipated lower prevalence of psychosis spectrum disorders and greater heterogeneity of case presentations in primary care relative to most previously explored settings, determining diagnostic accuracy is important to understanding the viability of primary care screening as a method to improving pathways to care for individuals with psychosis spectrum disorders. Mental health screeners in primary care are typically well received by patients, family members, and providers (Zuckerbrot et al., 2007), and can address barriers such as providers’ lack of time and training (French et al., 2012).

We evaluated the effectiveness of psychosis screening in on-site integrated behavioral health (IBH) services within eight primary care clinics, using the PQ-B. Participants completed an assessment of their symptoms via phone interview using the positive symptom subscale of the Structured Interview for Psychosis - Risk States (SIPS, Miller et al., 2003). This tool can assess both full psychosis and clinical high risk for psychosis (CHR), where individuals experience attenuated psychotic-like symptoms, functional impairment (Fusar-Poli et al., 2015), and are at elevated risk of developing full psychosis (Fusar-Poli et al., 2013). We hypothesized that the PQ-B would be an effective tool for differentiating individuals with psychosis spectrum disorder from those without a psychosis spectrum disorder.

## 2. Material and methods

### 2.1. Design

A prospective diagnostic accuracy study of screening for full psychosis and psychosis spectrum disorders in a IBH primary care setting was conducted utilizing the PQ-B (Loewy et al., 2011) as the index standard, and the SIPS (Miller et al., 2003) as the reference standard. All study-eligible individuals that attended the IBH appointment were offered the screening tool.

### 2.2. Participants

All individuals aged 18–30 attending an IBH intake assessment at one of six WellSpace Health primary care centers between 11/28/2017–03/23/2020 were potentially eligible for participation. In 05/2019, two WellSpace Health centers were added. Participants with a prior formal diagnosis of a psychotic disorder determined either via self-report or present in their electronic medical record, a documented IQ ≤70, or who were unable to use or felt uncomfortable using English at a level necessary to complete study assessments were excluded. Eligibility was assessed at the screening assessment stage and confirmed at the beginning of the phone assessment.

### 2.3. Settings

PQ-B screening was completed across eight WellSpace Health centers serving the Sacramento California area. As Federally Qualified Health Clinics (FQHCs), WellSpace Health centers provide comprehensive healthcare to all individuals regardless of their ability to pay, providing a critical health access point for those typically underserved.

Each center had a co-located IBH department where the individual receives their primary care services. Medical and behavioral health staff situated in the same building work closely together to provide collaborative care, with coordination between departments supported by care coordinators. The Wellspace IBH provides a range of services, including mental health assessments, individual and group counseling, substance use disorder treatment, family support, and psychiatry and medication management. WellSpace Health operates a low barrier model to IBH services, with referrals offered to all service users who either present or report behavioral health concerns, or score ≥ 5 on the Patient Health Questionnaire – 9 (PHQ-9; Kroenke et al., 2001) This was considered the most appropriate point to initiate psychosis screening, balancing the degree of reach against the likely prevalence of the target condition. The IBH services are staffed by licensed mental health clinicians and associate or bachelors-level care coordinators who coordinate the IBH intake process.

Follow-up assessments were conducted by the UC Davis (UCD) Early Psychosis (EP) Programs. The UCD programs serve individuals ages 12–30 who have experienced the onset of subthreshold or full psychosis in the past 2 years, serving individuals across commercial and state insurance funding.

### 2.4. Measures

The PQ-B (Loewy et al., 2011) is a 21-item self-report scale measuring positive psychotic-like experiences. The tool has been extensively validated as a screener for psychosis spectrum disorders (Savill et al., 2018), exhibits invariance across gender and race/ethnicity (Fonseca-Pedrero et al., 2018; Lång et al., 2021), and has been validated in many countries/languages (i.e., Fekih-Romdhane et al., 2023; Jang et al., 2019; Kaligis et al., 2018; Okewole et al., 2015).

For each endorsement, associated distress or impairment is rated on a 1–5 scale, 1 being “strongly disagree” and 5 “strongly agree”. These ratings, with a 0 for non-endorsed items, are summed for the distress score total. The tool is the first step in a two-step procedure, where individuals who score above a threshold then complete a full clinical interview. The PQ-B was designed to identify individuals experiencing attenuated psychotic symptoms but can also identify individuals with full psychosis (Rietdijk et al., 2012). A threshold of 18–24 has been validated in general community mental health clinics (Savill et al., 2018). Therefore, a provisional score of 20 was adopted to identify a positive screen in the current study. The distress total score, as opposed to the item score was adopted given this approach has been found to yield higher specificity (Loewy et al., 2011).

The reference standard used to determine the presence or absence of psychosis or psychosis spectrum disorder was a 90-min diagnostic phone interview based on the positive subscale of the Scale of Prodromal Symptoms (SOPS; Miller et al., 2003), conducted with both the participant and a collateral informant. The SOPS is the rating scale component of the SIPS, a validated diagnostic interview for CHR and threshold psychosis (Woods et al., 2019). The SOPS positive subscale assesses unusual thought content/delusional ideas, suspiciousness/persecutory ideas, grandiosity, perceptual abnormalities/hallucinations, and disorganized communication. Each item is rated 0–6, with a score of 3–5 within CHR range, and 6 at the level of full psychosis. Ratings are based on the duration, frequency, distress, conviction, and impairment of the experience. Additionally, we recorded possible contributory factors (e.g., trauma, substance use) to the reported symptoms. Psychosis spectrum disorder was defined as individual meeting criteria for either CHR or full threshold nonaffective or affective psychosis.

The phone assessors were BA level staff that conduct phone screen assessments as part of the referral process to the UCD EP clinics. All assessors received extensive training to reliability standards on the SIPS (v4.0) along with ongoing supervision and assessment review by licensed clinical psychologists. Following training, all assessors observed three phone assessments, and then were shadowed for an additional three before conducting assessments under supervision. In the parent study (Niendam et al., 2023), the SIPS syndrome diagnoses of the larger cohort (which included participants from this study) were found be 100 % consistent with the final intake assessment consensus diagnoses, and SIPS P-Scale rating reliability was also found to be excellent, with a mean test-retest correlation of *r* = 0.86.

On the referral form, for each positive screen, the referring clinician reported whether they agreed with the screening outcome, based on their clinical judgement. Responses were scored on a Likert scale of 1–5, 1 indicating they “disagree strongly”, and 5 “agree strongly”, with an additional option if they were “unsure”. This question was asked to determine how accurately primary care providers could identify psychosis spectrum disorders after the initial assessment based on clinical judgement.

### 2.5. Procedures

All IBH care coordinators and clinicians attended a 1-h provider education held by a licensed clinical psychologist. Topics included how to identify psychosis spectrum disorders, early intervention benefits, and UCD clinic and referral details. Each site received tablets with the study application, which included an informed consent, a demographic questionnaire, and the PQ-B (Loewy et al., 2011). Participants were then consented again at the phone screen stage.

Individuals were referred to the IBH department based on either clinical judgement of the primary care physician, or a PHQ-9 score ≥ 5 (Kroenke et al., 2001). Prior to the IBH clinic intake appointment, eligible participants completed study consent procedures and the PQ-B on a tablet. After completion, the tablet was returned to the provider. If the participants scored ≥20, the provider received the message “request phone interview from EDAPT” as a positive screen referral. If they scored ≤19, the provider received the message “continue to monitor or refer directly if still concerned”, and participants were invited to complete the same assessment as a negative screen research participant. Upon the participants’ agreement, the IBH provider submitted a referral to the UCD program, including whether they agreed with the positive screen outcome.

UCD staff called participants within one business day to schedule the phone assessment. After three failed attempts, the referring provider was contacted, and the participant was left one final voicemail. Phone interviews were completed by assessors blinded to the PQ-B score. If participants met the UCD program’s eligibility criteria, they were offered UCD clinic services. If they did not meet UCD criteria but still required specialty services, they were referred to appropriate care. If the person refused services or did not meet the specialty care threshold, they were referred back to their IBH provider. In cases where a psychosis spectrum disorder was not diagnosed, the assessor documented any relevant primary presenting behavioral health concerns. All research participants were compensated $50. Procedures were approved by the UCD IRB.

### 2.6. Data analysis

First, we examined the sample’s demographic characteristics to determine if any were related to the phone interview outcome. Next, we plotted receiver operating characteristic (ROC) curves to compare the PQ-B summary distress score to the dichotomous phone interview outcome classifications, including any appropriate sociodemographic variables as covariates. The area under the curve (AUC) for each ROC was calculated using the STATA [ROCREG] command. The AUC is statistically significant if the lower confidence interval is higher than 0.5, indicating accuracy beyond chance. Next, we calculated the sensitivity, specificity, positive and negative predictive value, positive and negative likelihood ratio, and diagnostic odds ratio (DOR) for various cut-off points to identify the most appropriate cutoff thresholds for use in primary care, selecting the value with the highest diagnostic odds ratio with a sensitivity <70 %. Once optimum cutoff thresholds were identified, we explored the clinical presentation of those who scored false-positively. Finally, the provider’s assessment of the PQ-B positive score as an indicator of psychosis spectrum disorder (CHR or full psychosis) was dichotomized with a provider response of “agree” or “strongly agree” rated a 1, and “unsure”, “disagree”, and “strongly disagree” rated a 0. The rationale for including “unsure” as a 0 was because it was considered unlikely that the provider would refer the participant to an EP program if they were unsure the client was experiencing psychotic-like experiences. The degree of congruency between this dichotomized outcome and the phone assessment diagnosis of psychotic spectrum disorder was compared using Cohen’s Kappa Statistic (κ).

## 3. Results

**Fig. 1.**
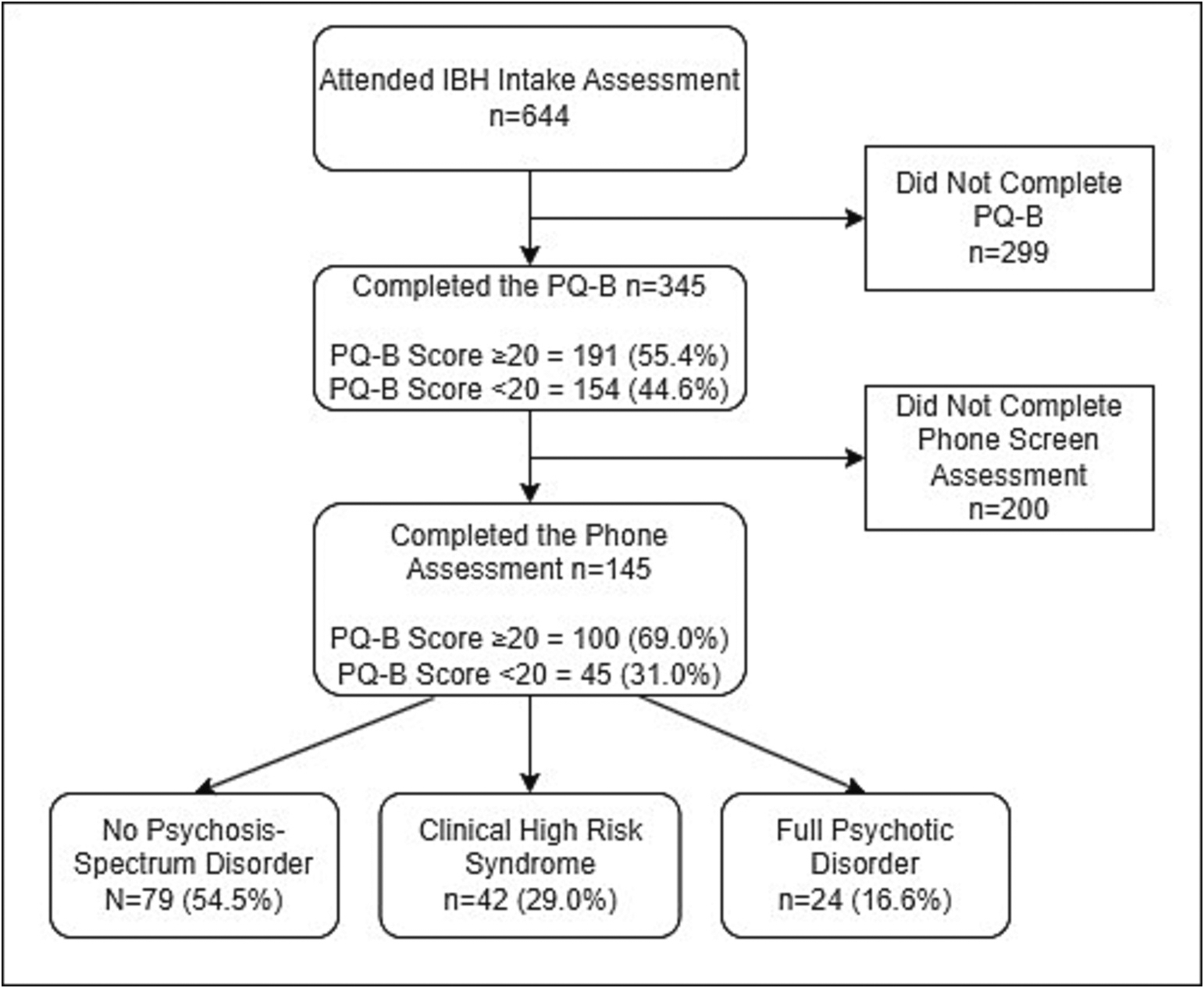
Study flow diagram. Key: IBH, Integrated Behavioral Health; PQ-B, Prodromal Questionnaire – Brief.

The STARD Flow diagram is presented in Fig. 1. From 11/28/2017–3/23/2020, 644 individuals aged between 18 and 30 attended an IBH intake assessment, of which 345 (53.6 %) completed the PQ-B. Of these, 191 scored ≥20 distress, and 154 below. One hundred individuals with ≥20 distress scores and 45 participants with ≤19 distress scores completed the phone assessment, resulting in a sample of 145.

In a comparison between those that did and did not score ≥ 20 PQ-B distress, no significant differences between age, gender, or ethnicity were detected. However, a significant difference across racial groups were detected (Chi^2^ = 14.57, *p* = .024), with a higher proportion of African American participants scoring ≥20 distress relative to other racial groups. Participants that scored ≥20 distress and attended the phone interview were slightly older than those that did not attend (*t* = −2.41, *p* = .017). Additionally, African American participants were significantly more likely to attend the phone interview after screening positively, relative to other racial groups (Z = 3.156, *p* < .001). Of those that attended the phone screen, 24 participants (16.6 %) met criteria for full psychosis, and 42 (29.0 %) met CHR criteria.

The demographics of the sample are presented in Table 1. In total, 72.4 % were female, with a mean age of 25.4 years. Approximately half identified as White (49.0 %), 16.5 % as African American, 4.1 % as Asian, 17.9 % identified as more than one race, and 11.0 % as “other”. Regarding ethnicity, 28.3 % identified as Hispanic/Latinx. In total, 60.7 % reported a household income below $35,000. A high proportion identified as LGBTQ+ (34.4 %). During the phone screen people identifying as Hispanic/Latinx, or more than one race were associated with a lower likelihood of being diagnosed with a psychosis-spectrum, so these variables were included as covariates in subsequent analyses.

**Fig. 2.**
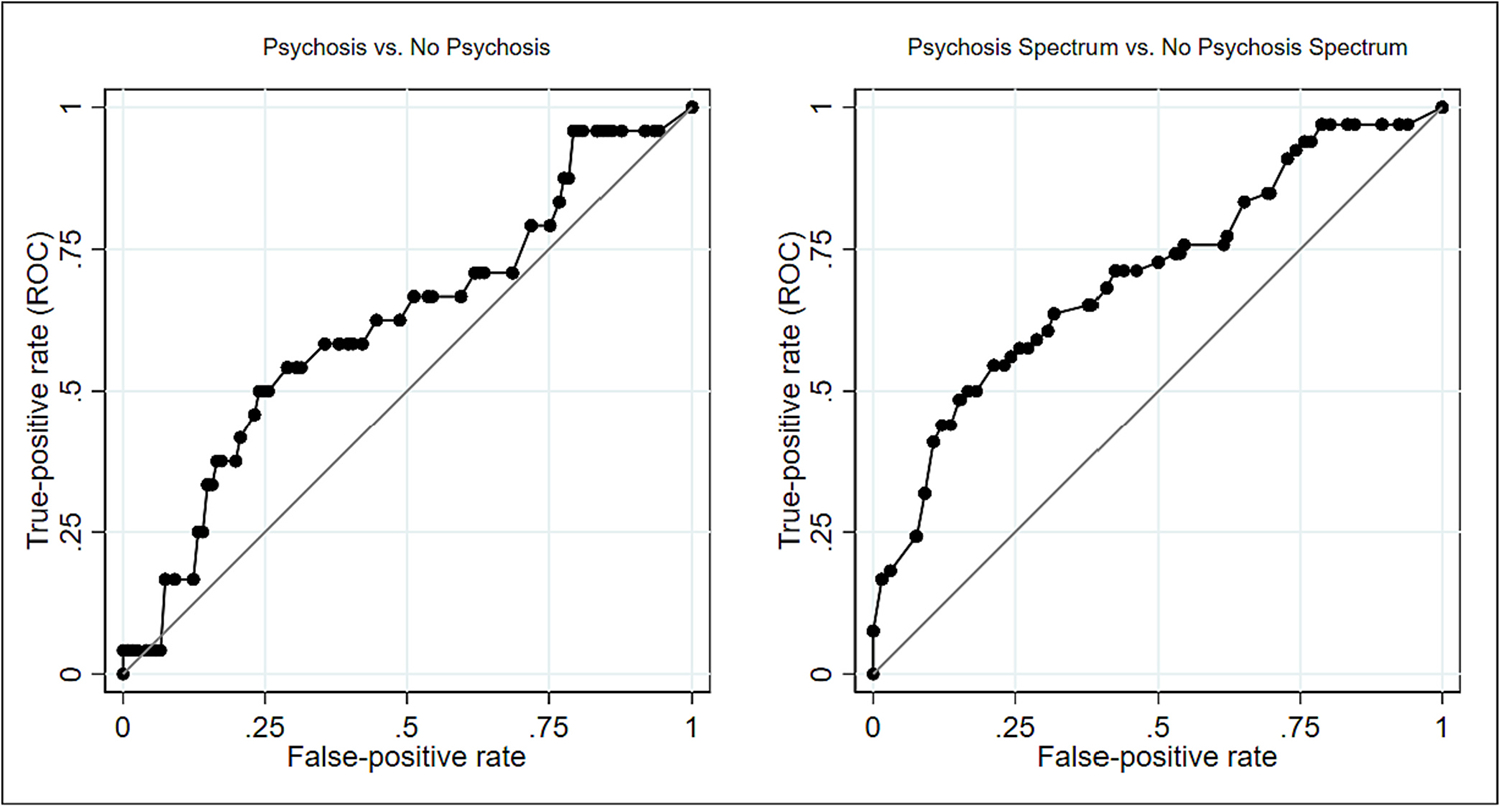
Receiver operating curves (ROC) indicating sensitivity and specificity of the Prodromal Questionnaire – brief in detecting psychosis and psychosis spectrum disorder.

The ROC curves presented in Fig. 2 detail the sensitivity and 1-specificity of the PQ-B total distress score compared to the SIPS. The PQ-B did not successfully predict a SIPS assessment of full psychosis versus no psychosis beyond chance (AUC 0.607, SE 0.065, 95 % CI 0.478 to 0.737). However, the PQ-B was effective at identifying individuals meeting criteria for a psychosis spectrum disorder (CHR and threshold psychosis), versus no psychosis spectrum disorder (AUC 0.693, SE 0.53, 95 % CI 0.590 to 0.796).

The accuracy statistics of different PQ-B distress score thresholds are presented in Table 2. In differentiating full psychosis from no psychosis, a PQ-B distress score of ≥24 had a sensitivity of 70.8 %, but a specificity of only 38.0 %. In differentiating psychosis spectrum disorder versus no psychosis spectrum disorder, a PQ-B distress score of ≥27 had the highest DOR above a sensitivity of 70 % (DOR = 3.27), with a sensitivity and specificity at 71.2 % and 57.0 % respectively. At the threshold of ≥27 to identify psychosis spectrum disorder, 47 were true positives, 45 true negatives, 34 false positives, and 19 false negatives. Amongst the 34 false positives, 31 individuals (91.2 %) indicated low mood, 30 (88.2 %) anxiety, 19 (55.9 %) had experienced trauma or crisis, 15 (44.1 %) were experiencing environmental stress, 13 (38.2 %) reported substance use, and 6 (17.7 %) reported a neurological condition.

IBH clinician impressions were available for 68 of 81 service users (84.0 %) that scored ≥27 distress. Amongst 10 cases where the providers either disagreed with the positive screen being an indicator of psychosis spectrum or were unsure, one was diagnosed with full psychosis, six participants were diagnosed as experiencing CHR, and three had no psychosis. Amongst the 46 cases where providers either agreed or strongly agreed with the positive screen as an indicator of psychosis spectrum disorder, nine were experiencing full psychosis, 15 were diagnosed with CHR, and 22 had no psychosis spectrum disorder. Overall, the degree of agreement between the IBH providers perspective of the PQ-B screening outcome and the phone screen assessment outcome was poor (48.2 % agreement, κ = −0.112).

## 4. Discussion

A PQ-B distress score of ≥27 detected psychosis spectrum disorder with a sensitivity of 71.2 % and a specificity of 57.0 %. This indicates the PQ-B is moderately effective at identifying individuals with psychosis spectrum disorder in IBH primary care settings, albeit less accurately than in more homogenous samples such as community mental health clinics and early psychosis clinical referrals (Savill et al., 2018). Linked to this, a higher threshold (≥27) was found to be needed, relative to the ≥24 value typically recommended in community settings (Savill et al., 2018). The relatively high prevalence of psychosis spectrum disorders amongst those screened (19.1 %) suggests that there may be sufficient cases in this mental health help seeking population to merit screening. In cases where the referring provider either disagreed with the positive screen or was unsure, 70.0 % were found to have CHR or full psychosis, highlighting the additive impact of screening all individuals that attend an IBH appointment and referring all who screening positively, over relying upon a provider’s clinical judgement to detect psychotic-like symptoms alone.

Regarding limitations, the sample was relatively small, albeit consistent with similar studies in the field (i.e., Savill et al., 2018). Additionally, a high dropout rate was observed between assessment stages, consistent with the parent study (i.e., Niendam et al., 2023). Approximately half of all IBH service users aged between 18 and 30 did not complete the PQ-B (53.6 %). Unfortunately, no data was available to determine what proportion of this total was not eligible, elected not to participate, or were not offered the tablet due to service-level factors. However, it is possible that those who do not experience psychotic-like experiences would be less likely to agree to the screen, leading to an enriched sample. Linked to this, the finding that 55.4 % scored ≥20 is slightly higher than the figure seen in the parent study (46.5 %) (Niendam et al., 2023), highlighting the high degree of distress experienced amongst those in this sample. Additionally, almost half (47.6 %) of individuals who were referred to UCD after scoring ≥20 did not attend the phone screen. As a result of this dropout, caution should be exercised in interpreting the high proportion of those identified as having a psychosis spectrum disorder in the phone screen stage (45.5 %), given it is likely that those that did not meet this diagnosis were disproportionately more likely to drop out in prior stages.

The high study dropout suggests the need to further support the busy, and often under-resourced IBH primary care setting for screening implementation, engagement, and linkage (Woodberry et al., 2022). Incorporating screening into the electronic medical record could result in more inclusive screening, as has been done with PHQ9 screening for depression (Jetelina et al., 2018). Other methods of engagement, such as the use of text, may also be beneficial (D’Arcey et al., 2020). Qualitative research exploring why individuals drop out throughout the process could also help to identify strategies to minimize barriers to specialty care for those that need it. Notably, the major exception to the high drop out at the phone screen stage was amongst African American participants, where 79.3 % of those who screened positively completed the phone screen. While there is research to suggest the primary care setting may not be a frequent point of contact in the pathway to care in early psychosis amongst Black and African Americans (Anderson et al., 2014; Compton et al., 2006; Oluwoye et al., 2021), the finding that African American participants are more receptive to seeking services following a positive PQ-B screen has been detected previously (Savill et al., 2022), suggesting screening may have a part to play in addressing longstanding inequities in community mental health care engagement in the US (Creedon and Cook, 2016).

Notably, PQ-B screening was completed during the on-site IBH intake appointment. Consequently, this study cannot determine the diagnostic accuracy of the PQ-B amongst all those that attend a primary care appointment, which does limit the generalizability of the findings. WellSpace Health leadership considered this the most viable stage to implement the screening process given the short length of typical primary care appointments and lower expected prevalence of psychotic spectrum disorders. It is possible that some psychosis-spectrum individuals were not referred from the primary care appointment to IBH, meaning some cases remained unidentified. Due to the low barrier threshold adopted to trigger a referral we did not anticipate this to represent many individuals, but this question remains unanswered.

Amongst individuals who scored high on the PQ-B but did not have a psychotic spectrum disorder, almost all exhibited signs of psychological distress with multiple factors that may attribute to such experiences. These include experiences of trauma, substance use, low mood, and anxiety. These high rates may be a feature of the sample, which was predominantly low income, in addition to highly racially diverse and LGBTQ+, which can lead to increased minority stress (Cyrus, 2017). Regardless, these findings highlight an important issue: a false positive screen on the PQ-B does not necessarily indicate that the person is not in need of behavioral health services. Therefore, if organizations are considering screening for psychosis spectrum disorders, it is important to clearly identify care pathways for individuals who might not meet criteria for early psychosis care but still require mental health services.

Notably, when the provider disagreed or was unsure of the positive screen being indicative of a psychosis spectrum disorder, the participant was diagnosed with CHR or full psychosis in 70 % of cases. The sample size was low (*n* = 10) meaning caution should be exercised. However, this occurred despite the additional training providers received around psychosis symptom identification, suggesting discrepancies could be even higher in usual care where such training is rare. Furthermore, it is possible that this degree of difference may have been even higher were the clinician blinded to the screening outcome, which was not possible due to study procedures. The intake assessments completed in the IBH department were relatively brief (30–50 min). It is unclear if these clients would be later identified if providers had more time to explore their psychopathology. Other studies suggest many people with psychosis spectrum disorders seen in primary care and community mental health settings are likely not identified, or not referred to specialty care promptly, thus incurring DUP delays (Anderson et al., 2013b; Rietdijk et al., 2012). Given the critical importance of early intervention in improving outcomes in psychosis (Kane et al., 2016; Perkins et al., 2005), and reducing transition from CHR to full psychosis (Fusar-Poli et al., 2013; Raballo et al., 2019) this finding highlights the potential impact that screening in primary care settings could have.

Finally, while it was notable that the PQ-B was effective at identifying individuals with psychosis spectrum disorder, it was not effective at identifying cases with full psychosis only. This is unsurprising, given the PQ-B was designed to identify those experiencing attenuated psychotic experiences and so would likely lead to many CHR cases being defined as false-positives. In some regions or states such as California, specialty psychosis programs typically treat both full psychosis and CHR (Niendam et al., 2019). In situations where screening is focused exclusively on identifying individuals with full psychosis, future work may be necessary to validate alternative assessment tools.

### 4.1. Conclusions

With a sensitivity of 71 % and specificity of 57 %, the PQ-B was only moderately successful at identifying individuals with psychosis-spectrum disorders in the IBH primary care setting. However, these findings do suggest that the PQ-B can effectively identify individuals with psychosis spectrum disorder in an IBH primary care setting, can identify people that might otherwise be missed based on clinical judgement alone, and that the prevalence of psychosis spectrum disorders in this setting may be sufficient to merit such efforts. With the ongoing expansion of CHR services in the United States, many funded by the Substance Abuse and Mental Health Services Administration, such screening efforts may represent an important pathway to ensuring those in need can receive such care.

## Supplementary Material

supplementary data

## Figures and Tables

**Table 1:** Demographic details of the sample.

Variable	*n* = 145	

WellSpace Health Site (n, %)		
Alhambra	34	23.45
Sunrise	28	19.31
San Juan	27	18.62
J. St	24	16.55
South Valley	14	9.66
Norwood	12	8.28
Rancho Cordova	5	3.45
Arden	1	0.69
Sex (n, %)		
Male	40	27.6 %
Female	105	72.4 %
Age (Mn, SD)	25.4	3.11
Race (n, %)		
Caucasian	71	48.97
African American/Black	24	16.55
Asian	6	4.14
> 1 race	26	11.03
Other	16	12.0
missing	2	1.38
Ethnicity (n, %)		
Hispanic	41	28.28
Non-Hispanic	102	70.34
missing	2	1.38
Sexual Orientation/Gender Identity		
Heterosexual	94	64.83
LGBTQ+	47	32.41
Other	4	2.76
Annual Household Income (n, %)		
<$5000	7	4.83
$5000 - $11,999	12	8.28
$12,000 - $15,999	15	10.34
$16,000 - $24,999	26	17.93
$25,000 - $34,999	28	19.31
$35,000 - $49,999	20	13.79
$50,000 - $74,999	9	6.21
$75,000 - $99,999	7	4.83
$100,000+	4	2.76
Missing	17	11.72
PQ-B Total Distress (Mn, SD)	31.28	21.01

Key: LGBTQ+: Lesbian, Gay, Bisexual, Transgender, Queer +; Mn: Mean; PQ-B: Prodromal Questionnaire - Brief; SD: Standard Deviation.

**Table 2:** Diagnostic accuracy of Prodromal Questionnaire - brief as a screener for psychosis spectrum disorder in a primary care setting.

Sensitivity		Specificity	PPV	NPV	LR+	LR-	DOR

Full Psychosis vs. No Full Psychosis							
Distress Score ≥ 18	79.2 %	28.1 %	17.9 %	87.2 %	1.10	0.74	1.49
Distress Score ≥ 19	75.0 %	28.1 %	17.1 %	85.0 %	1.04	0.89	1.17
Distress Score ≥ 20	70.8 %	31.4 %	17.0 %	84.4 %	1.03	0.93	1.11
Distress Score ≥ 21	70.8 %	36.7 %	18.1 %	86.3 %	1.11	0.80	1.39
Distress Score ≥ 22	70.8 %	37.2 %	18.3 %	86.5 %	1.13	0.78	1.44
Distress Score ≥ 23	70.8 %	38.0 %	18.5 %	86.8 %	1.13	0.78	1.49
Distress Score ≥ 24	70.8 %	38.0 %	18.5 %	86.8 %	1.14	0.76	1.49
Distress Score ≥ 25	66.7 %	40.5 %	18.2 %	86.0 %	1.12	0.82	1.36
Psychosis Spectrum vs. No Psychosis Spectrum							
Distress Score ≥ 18	83.3 %	35.4 %	51.9 %	71.8 %	1.29	0.47	2.75
Distress Score ≥ 19	81.8 %	35.4 %	51.4 %	70.0 %	1.27	0.51	2.47
Distress Score ≥ 20	77.3 %	38.0 %	51.0 %	66.7 %	1.25	0.60	2.08
Distress Score ≥ 21	75.8 %	44.3 %	53.2 %	68.6 %	1.36	0.55	2.49
Distress Score ≥ 22	74.2 %	44.3 %	52.7 %	67.3 %	1.33	0.58	2.29
Distress Score ≥ 23	74.2 %	45.6 %	53.3 %	67.9 %	1.33	0.58	2.41
Distress Score ≥ 24	74.2 %	45.6 %	53.3 %	67.9 %	1.36	0.57	2.41
Distress Score ≥ 25	72.7 %	49.4 %	54.5 %	68.4 %	1.44	0.55	2.60
Distress Score ≥ 26	71.2 %	55.7 %	57.3 %	69.8 %	1.61	0.52	3.11
Distress Score ≥ 27	71.2 %	57.0 %	58.0 %	70.3 %	1.65	0.51	3.27
Distress Score ≥ 28	68.2 %	58.2 %	57.7 %	68.7 %	1.63	0.55	2.99

Key: PPV: Positive predictive value, NPV: Negative predictive value, LR: Like-lihood ratio, DOR: Diagnostic odds ratio.
