# Supplementary figures and images for: The diagnostic accuracy of screening for psychosis spectrum disorders in behavioral health clinics integrated into primary care
Source: Schizophr Res. Author manuscript; Available in PMC 2024 May 31. (PMC11140870; doi:10.1016/j.schres.2024.02.007)

### Supplemental Figure 1: Study Procedures


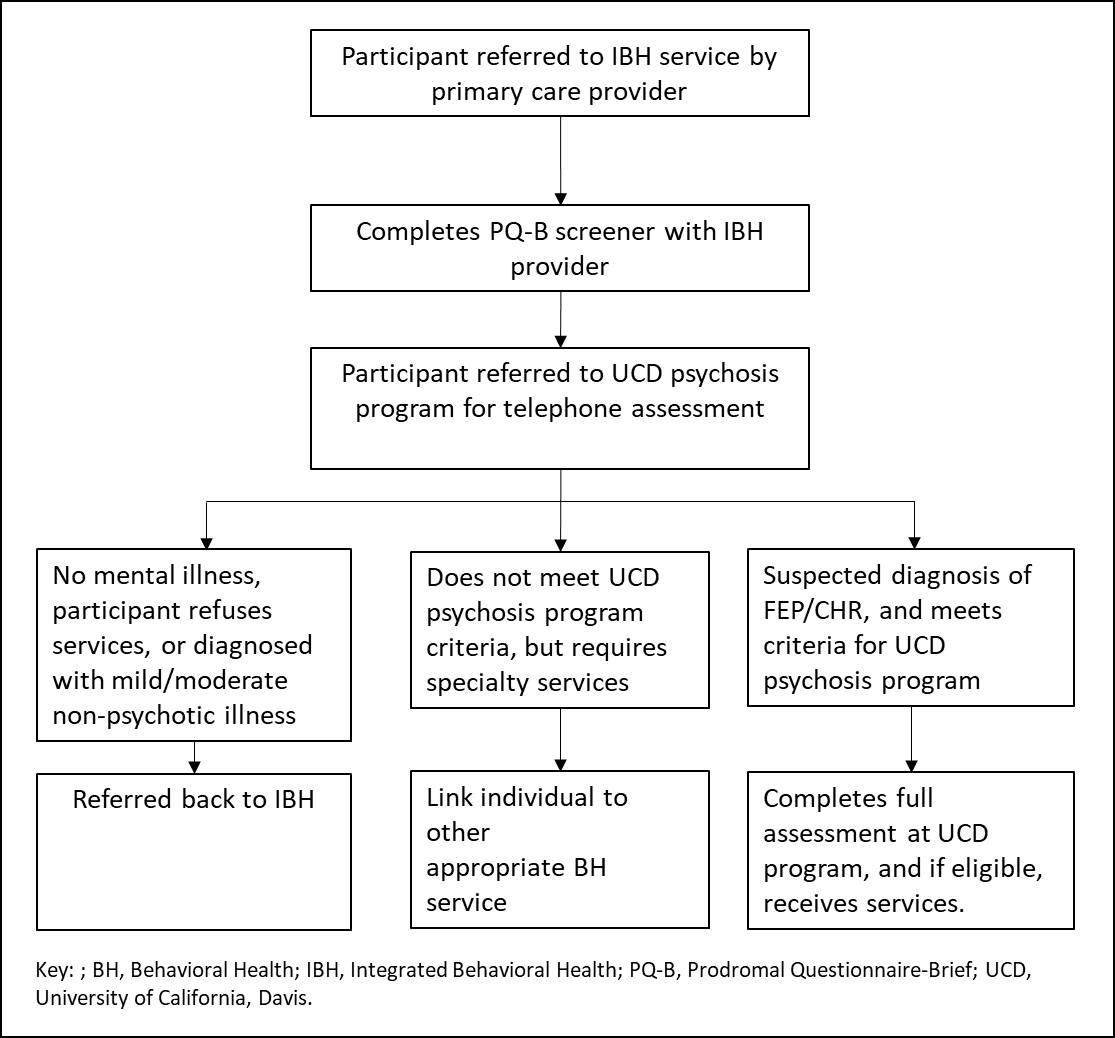

Supplement: supplementary data [file NIHMS1994001-supplement-supplementary_data.docx]
